# Intravenous Ascorbic Acid and Lung Function in Severely Ill COVID-19 Patients

**DOI:** 10.3390/metabo12090865

**Published:** 2022-09-14

**Authors:** Sara Sokary, Asma Ouagueni, Vijay Ganji

**Affiliations:** Human Nutrition Department, College of Health Sciences, QU Health, Qatar University, Doha P.O. Box 2713, Qatar

**Keywords:** acute respiratory distress syndrome, ascorbic acid, COVID-19, critical illness, lung function, ICU, viral infection, vitamin C

## Abstract

Current evidence suggests that ascorbic acid improves the host’s immune system and, therefore, may play a role in reducing the severity of infectious diseases. Coronavirus disease 2019 (COVID-19) is a potentially life-threatening viral infection that mainly infects the lungs. The objective of this review was to synthesize the existing findings from studies related to the effect of intravenous ascorbic acid on lung function in COVID-19 patients. For this review, PubMed, Cochrane, SCOPUS, EMBASE, Clinical Trial Registry, and Google Scholar databases were searched from December 2019 to May 2022. There was a total of six studies that investigated the large dose of ascorbic acid infusion intravenously on lung function in severely ill subjects with COVID-19. Out of six, three studies found that high-dose intravenous ascorbic acid improved lung function markers, and three studies found null results. Infusions of 12 g/d and 24 g/d of intravenous ascorbic acid had shown a significant improvement in lung function markers in two clinical trials. Studies that administered 8 g/d, 2 g/d, and 50 mg/kg/d of intravenous ascorbic acid found no influence on mechanical ventilation need and other lung function markers in critically ill subjects with COVID-19. Overall, the effect of intravenous ascorbic acid on the lung function of subjects with COVID yielded equivocal findings. More double-blinded, randomized, clinical studies with a larger sample size are required to confirm the effect of ascorbic acid in ameliorating the lung pathologies associated with COVID infection.

## 1. Introduction

The novel coronavirus disease 2019 (COVID-19) is a potentially fatal viral infection caused by severe acute respiratory syndrome coronavirus 2 (SARS-CoV-2). According to the WHO report, COVID-19 resulted in more than 510 million cases and 6.2 million deaths by May 2022 [1]. The coronaviruses affect the respiratory and digestive systems, and the symptoms are generally considered mild in many patients. In some patients, the effects of COVID-19 may be seen for months after the active infection has been cured [2,3,4]. The initial symptoms of COVID-19 are cough, fatigue, fever, diarrhea, and headache [5]. The most common symptom of severe COVID-19 infection is dyspnea usually accompanied by hypoxemia, leading to the development of progressive respiratory failure or acute respiratory distress syndrome (ARDS) [6]. Patients with ARDS experience severe hypoxemia, lung edema, and acute onset of bilateral infiltrates [7]. More serious complications are multiple organ dysfunction (acute cardiac, liver, and kidney injury) that can lead to shock and death [8]. Several factors such as age, micronutrient deficiency, and chronic diseases can affect the severity of the symptoms of COVID-19 [6,9,10,11]. Older men and patients with multiple comorbidities were more likely to be admitted to intensive care unit (ICU) and on mechanical ventilation [8]. Therefore, treatments mainly focus on the management of respiratory-related dysfunction.

Current treatments that are available for COVID-19 aim at ameliorating the symptoms. The American Thoracic Society and European Respiratory Society have jointly issued guidelines for the management of COVID-19 in hospitalized patients with lung dysfunction symptoms [12]. Several tests are used for the detection of lung function status. These include the ratio of forced expiratory volume in the first second to inspiratory vital capacity, diffusion capacity for carbon monoxide, and maximal expiratory flow rate at 25% of vital capacity [13]. Other tests such as partial pressure of oxygen to the fraction of inspired oxygen (PaO_2_/FiO_2_), which indicates oxygenation status and blood oxygen saturation (SaO_2_), are also indicators of lung function. Severe COVID-19 cases are usually managed by placing patients on mechanical ventilation to improve respiration due to the ARDS [13]. Findings from several studies indicated that micronutrients in general [14], specifically ascorbic acid, may be beneficial in the prevention and treatment of symptoms associated with COVID-19 [15,16,17]. Since ascorbic acid has an immune-modulatory function, administration of ascorbic acid to critically ill patients may improve the lung pathologies associated with COVID-19 infection. It is not clearly known if a large dose of intravenous infusion of ascorbic acid would improve the lung function of severely ill patients with COVID. Therefore, the current review aimed to summarize and synthesize the current literature on the effect of intravenous administration of ascorbic acid on lung function in critically ill patients with COVID-19.

## 2. Literature Search Strategy

We searched several databases such as the Cochrane Library, EMBASE, PubMed (Medline), SCOPUS, Clinical Trial Registry, and Google scholar for scientific papers published through April 2022. The following key terms were used: (“ascorbic acid” OR “vitamin C” OR “Sodium Ascorbate” OR “L-ascorbic acid”) AND (“coronavirus” OR “COVID 19” OR “COVID-19” OR “COVID” OR “SARS-CoV2”) AND (“Intensive Care Unit” OR “ICU” OR “Severe” OR “Critically”). 

## 3. Functions of Ascorbic Acid

Ascorbic acid is a hydrophilic vitamer that needs to be obtained from dietary sources, as it is not synthesized in the human body due to mutations in the gene responsible for producing gluconolactone oxidase, the last enzyme in the biosynthesis of ascorbic acid [18]. Ascorbic acid is involved in many biochemical functions. These include collagen synthesis and other multiple functions that are beneficial in critical illness such as antioxidant, anti-inflammatory, stimulant of nor-adrenaline, vasopressin synthesis, anticoagulant, and immune-modulatory functions [19]. To maintain health and prevent ascorbic acid deficiency-induced scurvy, it is recommended to have a daily intake of 90 mg for men and 75 mg for women [19]. This amount might not be sufficient in cases of critical illness requiring intensive care [20]. Furthermore, in critically ill patients, due to increased need, circulating ascorbic acid concentrations rapidly decline unless higher doses are given [21]. In a viral infection, the requirements of ascorbic acid range from 2 g to 3 g/d to maintain normal plasma ascorbic acid concentrations between 60 and 80 μmol/L [22,23]. However, studies have shown that up to 8 g/d might be needed during high physiological stress [24,25]. In fact, the National Institutes of Health expert panel has recommended up to 1.5 g/kg body weight, an amount that presents as safe without significant deleterious effects [26]. However, the high plasma concentration cannot be achieved orally, as a high dose of oral ascorbic acid saturates the intestinal absorption mechanism [27]. Therefore, higher plasma concentration can be achieved through intravenous infusion of ascorbic acid. A 200 mg daily oral dose produced a plasma concentration of 70 to 90 μmol/L of circulating ascorbic acid in healthy individuals [28,29]. However, the same dose that was given intravenously raised plasma ascorbic acid concentrations by ≈10-fold [19]. Because ascorbic acid has immunomodulatory [30], anti-inflammatory [31], and antioxidant properties [32], this vitamer has been studied for its potential role in the prevention and treatment of multiple viral diseases, including COVID-19 [33,34,35,36]. Ascorbic acid is widely used in the clinical setting due to its low cost and high safety [37]. Moreover, several clinical trials have established the efficacy and safety of high-dose intravenous ascorbic acid (HDIAA) in critically ill patients [38,39,40].

## 4. Metabolism of Ascorbic Acid

After the ingestion of foods with ascorbic acid, the majority of it is absorbed through the intestinal epithelium by a family of transporter proteins [41]. Absorption of ascorbic acid occurs through the active transport system [42]. The distribution of ascorbic acid in the tissues is highly compartmentalized. The brain and nervous system contain the highest concentration [42,43,44]. Under normal physiological conditions, ascorbic acid undergoes sequential steps to form various metabolites [44]. Ascorbic acid functions as a reducing agent by donating electrons. This vitamer is mostly present in the form of ascorbate anion [45]. First ascorbic acid is oxidized by losing one electron to form an unstable ascorbate radical. After less than one millisecond, another electron is lost, leading to the formation of a more stable oxidized dehydroascorbic acid (DHA) [46]. This DHA can be reversibly reduced back to ascorbic acid. These two forms of vitamers are transported in circulation by different transporters, i.e., sodium vitamin C transporter (SVCT) and glucose transporter (GLUT) [47,48]. The former transporter family, SVCT1 and SVCT2, is responsible for delivering ascorbic acid to the cells. SVCT1 is primarily located in absorptive cells and the liver [45]. SVCT2 is located on the remaining body tissues [49]. GLUT 1, 2, 3, 4, and 8 are responsible for transporting DHA to the cells [50,51,52]. Further DHA is hydrolyzed to 2,3-diketo-l-gulonate, which is later split to form L-erythrulose and oxalate. These metabolites and ascorbic acid are then filtered efficiently by the hydrostatic pressure gradient in the proximal convoluted tubule in the kidneys. Under deficiency conditions, ascorbic acid reabsorption is achieved by SVCT1 transporters. However, when the body is saturated with ascorbic acid, surplus vitamin excretion occurs through the kidneys [28]. Consequently, in susceptible individuals, the oxalate formed as a result of ascorbic acid catabolism may contribute to the formation of kidney stones [53]. A recent scoping review showed that among 2801 participants receiving HDIAA, five cases of oxalate nephropathy and one case of kidney stones were reported [54].

## 5. Effect of Ascorbic Acid on Lung Function

Ascorbic acid has been reported to play a protective role in lung function. Oxygen free radicals damage phospholipids in the cell membrane, proteins, nucleic acids, and extracellular matrix [55]. As an antioxidant, ascorbic acid protects against cellular damage by scavenging reactive oxygen species (ROS) by rapid electron transfer. Thus, oxidative stress and oxidative damage are lowered in the body and enhance recovery after critical illness [37,56]. Moreover, ascorbic acid is the most abundant water-soluble vitamin in the extracellular fluid of the lung [57], especially in alveolar macrophages and alveolar type II cells [58], which contain almost 30 times higher ascorbic acid than the blood [59]. These high concentrations may imply a role for ascorbic acid in lung function.

Previous studies showed that ascorbic acid upregulates the production of prostaglandin I_2_ in the airway, which leads to pulmonary vasodilation [60]. Additionally, ascorbic acid ameliorates structural damage in the alveoli and pulmonary blood vessels [61]. The effect of ascorbic acid was confirmed in an ex vivo model by an independent interaction with protein complexes in the electron transport chain to stabilize the function of the isolated lung by preserving mitochondrial activity [62]. Furthermore, ascorbic acid was found to reduce capillary–alveolar damage and mortality in restraint-stressed mice with H1N1 viral-induced pneumonia [63]. This explains the possibility of ascorbic acid in improving the clinical status of COVID-19 patients. A recent meta-analysis assessed the effectiveness of high doses of ascorbic acid on mechanical ventilation days, hospital length of stay, and ICU administration. This study showed that ascorbic acid administration reduced mechanical ventilation days significantly [64].

## 6. Effect of COVID-19 on Lung Function 

The pathology of COVID-19 is similar to SARS or MERS infections. Although COVID-19 infection affects multiple organs, it mostly affects the lungs. The possible mechanism of COVID-19 infection starts with the binding of cellular angiotensin-converting enzyme 2 (ACE2) to SARS-CoV-2 spike (S) protein as well as to other cell membrane proteins [65,66]. The S protein is composed of two subunits, which are S1 and S2, where the former is required for binding to host cell receptors and the latter mediates the fusion of viral and host cellular membranes [67,68,69]. The SARS-CoV-2 viral replication is associated with the production of pro-inflammatory cytokines along with causing cell apoptosis [70]. The overproduction of cytokines is called a cytokine storm, a severe inflammatory response by the host’s immune system [71]. It generates uncontrollable and vigorous inflammatory reactions that increase the pro-inflammatory cytokines [72]. It is proposed that the cytokine overproduction is mediated by reduced ACE2 surface expression due to internalization after the attachment with the S protein [73]. The ACE2 protein is most abundant in alveolar epithelial cells and enterocytes. This explains why the lungs are damaged first in SARS-CoV-2 infection [74,75]. The increased cytokine concentration damages the alveolar capillaries [76]. This results in interstitial edema and filling up with alveolar fluid [77], leading to low oxygen saturation and arterial pressure and an increased need for mechanical ventilation.

## 7. Effect of Intravenous Ascorbic Acid on the Lung Function of Severe COVID-19 Patients

### 7.1. HDIAA Intravenous Infusion Studies

Multiple studies have examined the effects of high doses of ascorbic acid on the prognosis of COVID-19 patients, but few studies focused on the improvement of lung function. The summary of studies on the effect of the administration of intravenous ascorbic acid on the lung function of severally ill COVID-19 patients is presented in Table 1. As the COVD-19 pandemic started, a multicenter pilot RCT by Zhang et al. was the first to publish results on the effect of HDIAA on lung function in severe COVID-19 patients [33]. The study assessed lung function through invasive mechanical ventilation-free days over 28 days (IMVFD28), and secondary outcomes related to lung function were invasive mechanical ventilation (IMV), high flow nasal cannula (HFNC), and non-invasive mechanical ventilation (NIMV) days over 28 days. In total, 27 participants admitted to the ICU received 12 g/50 mL of ascorbic acid every 12 h (total 24 g/day) for 7 days, while 29 served as a control. The findings showed a significant improvement in PaO_2_/FiO_2_ ratio (*p* = 0.01 ) and IL-6 after HDIAA (*p* = 0.04). Further, a decrease in IMVFD28 was observed, however, the difference was not significant between the intervention and the control groups (*p* = 0.57, HR: 4.8, CI: −4.7 to 7.2). All other secondary outcomes were not improved by HDIAA (IMV: *p* = 0.6, HFNC: *p* = 0.85, and NIMV: *p* = 0.68). Notably, administration of an HDIAA caused a trend toward reduction in the 28-day mortality in patients with severe symptoms (*p* = 0.06). It is important to note that this study included a small number of participants to generate enough power for the intended study design. Another retrospective cohort by Gao et al. included 46 severe COVID-19 patients [78]. The intervention was a 12-g intravenous infusion on the first day, then 6 g/d for the following 4 days. Relating to lung function, oxygen support status was significantly enhanced in the intervention group compared to the control group (63.9% vs. 36.1%). They defined the improvement in oxygen support status as a decrease on the seven-category ordinal scale by at least two points from baseline until discharge on day 28.

### 7.2. Medium/Low-Dose Ascorbic Acid Intravenous Infusion Studies

In an open-labeled, non-blinded RCT, investigators infused 2 g of HDIAA for 30 ICU COVID-19 patients [79]. In lungs, peripheral capillary oxygen saturation (SpO_2_) was significantly improved after three days of infusion of HDIAA (*p* = 0.014). However, this improvement was not observed at discharge. Further, the mean body temperature on day third improved significantly (*p* = 0.001). The findings of this study are consistent with Zhang et al. [33], where a similar improvement in PaO_2_/FiO_2_ was seen. Kumari et al. conducted an open-label RCT [35] that examined the need for mechanical ventilation (they excluded patients that required mechanical ventilation within 12 h of admission). The intervention was an administration of 50 mg/kg/d of intravenous ascorbic acid in 150 severe COVID-19 patients. The findings showed no difference in need for mechanical ventilation between treatment and control groups (*p* = 0.41) but pointed to a beneficial effect in reducing the days of hospitalization and days to become symptom-free. In addition, a retrospective study [34] included 323 patients with ARDS associated with COVID-19. These patients were subsequently categorized as patients that received HDIAA and controls. The intervention group consisted of 153 patients that received 2 g/d intravenous ascorbic acid for 3 days. The results showed no difference between the patients that received HDIAA and controls for all outcome measurements. Specifically, for lung function, the need for advanced oxygen support was similar in both groups (*p* = 0.49). Another study assessed the effect of high doses of ascorbic acid, melatonin, and zinc on ARDS due to COVID-19 infection [80]. Standard care plus 8 g/d of intravenous ascorbic acid were given to 10 adults for 10 days, in addition to 6 mg of oral melatonin and 50 mg of oral zinc sulfate every 6 h. This study aimed to examine changes in hypoxemia and inflammatory markers as primary outcomes. Investigators reported that SaO_2_ and PaO_2_/FiO_2_ were improved in both the intervention and the control groups. The limitation of this study was the small sample size. Overall, administering medium/low doses of intravenous ascorbic acid was not effective in improving the condition of COVID-19 patients compared to high doses. 

Notably, a double-blinded RCT is awaiting completion, with a parallel design of 80 patients in each of the intervention and control groups. The study will measure oxygenation level (oxygen saturation or oxygen flow) in non-ventilated patients as well as PaO_2_/FiO_2_ ratio in ventilated patients as primary outcomes and indicators for pulmonary function after administration of HDIAA (NCT05029037). This study is expected to provide the latest evidence regarding the effect of HDIAA on severe SARS-CoV-2 infections.

## 8. Plausible Mechanisms of Ascorbic Acid in Improving Severe SARS-CoV-2 Infection

Because ascorbic acid is an immune-protective agent, it is predicted to protect against the exacerbation of symptoms during the critical phase of SARS-CoV-2 infection. The immunomodulation effects of ascorbic acid are due to its antioxidant properties as it takes part in the redox reactions, where it gets oxidized to form DHA [81]. Studies conducted before the COVID-19 pandemic have shown that DHA exerts antiviral effects, as it was found to inhibit replication of poliovirus type A, influenza virus type A, herpes simplex virus type 1, and rabies influenza [82]. Furthermore, ascorbic acid administration is found to restore the epithelial barrier in the lungs, which aids in improving severe infections [83] with immune-regulatory properties [84].

**Figure 1:**
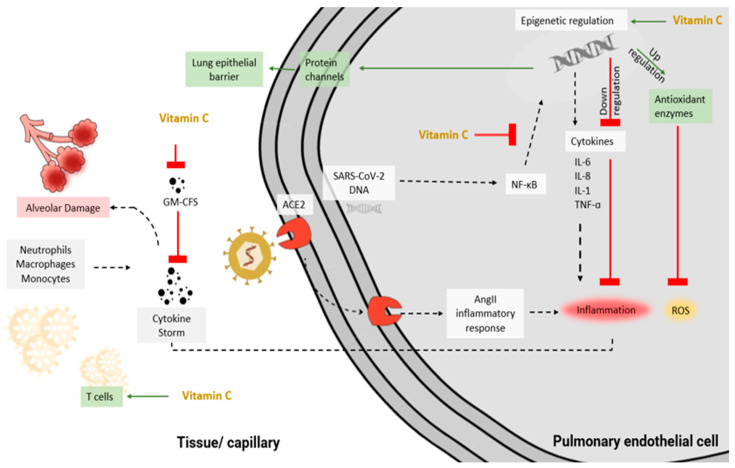
Anti-inflammatory and anti-viral mechanisms of ascorbic acid in SARS-CoV-2 infection. Abbreviations: ACE2, Angiotensin-converting enzyme 2; AngII, Angiotensin-II; GM-CFS, Granulocyte–macrophage-colony-stimulating factor; IL, Interleukin; NF-κB, Nuclear factor kappa-B; ROS, Reactive oxygen species; TNF-α, Tumor necrosis factor-alpha.

It has been reported that ascorbic acid aids in the downregulation of cytokines in severe COVID-19 infection, which protects the endothelium from oxidant injury and is essential for tissue repair [85,86]. Activation of the nuclear factor kappa-B (NF-κB), a primary pro-inflammatory transcription factor, regulates the interaction between oxidative stress and the genes that encode inflammatory markers such as TNFα, IL-1, and IL-8 [87]. Because ascorbic acid has been reported to attenuate NF-κB, this results in lower ROS and inflammation [88] (Figure 1).

Additionally, an in vivo investigation showed that ascorbic acid increases the antioxidant enzymes such as catalase, glutathione, and superoxide dismutase and decreases serum pro-inflammatory cytokines (TNFα and IL-1β) and their mediators in a dose-dependent manner [88,89]. Nonetheless, these mechanisms may be mostly due to the epigenetic regulation of ascorbic acid on various genes, i.e., upregulating antioxidant proteins and downregulating pro-inflammatory cytokine production. The epigenetic and transcriptional regulation results in enhanced protein channels that regulate alveolar fluid clearance, leading to improved lung epithelial barrier function, respiratory function, and ARDS status [83,90]. An animal study showed that parenteral infusion of ascorbic acid to mice with acute lung injury had a protective effect on lung function by improving alveolar fluid clearance, preventing vascular injury, restoring endothelial and alveolar epithelial integrity, and augmenting lung barrier cell function by modulating ROS generation and expression, thus preventing ROS-induced lung damage [83,91]. Evidence also showed that ascorbic acid reduces the signaling response of the granulocyte–macrophage-colony-stimulating factor, a cytokine that has a major role in the host’s autoimmune and inflammatory response to pulmonary infection [92]. 

Innate immunity is influenced by the regulation of neutrophil function [84]. The extent of tissue damage is reduced by influencing the neutrophil apoptotic process in the inflammation pathway [17,93] and by reducing the formation of extracellular trap nets [94]. A high dose of ascorbic acid is found to play a vital role in the regulation of the proliferation and function of natural killer cells, B cells, and T cells [95,96]. COVID-19 results in the dysfunction of T cells [97]. Recent research showed that T-cell maturation is enhanced by the epigenetic properties of ascorbic acid [98]. This process explains the role of ascorbic acid in viral clearance via T-cells. 

## 9. Limitations, Summary, and Conclusions 

Thus far, the evidence available on the effect of a HDIAA in improving the lung function of severe COVID-19 patients is promising. To achieve these beneficial effects, a dose of at least 12 to 24 g/d is required, based on the limited evidence. Therefore, the dosage is an important consideration in intravenous ascorbic acid therapy in severely ill COVID-19 patients. Medium to low doses of intravenous ascorbic acid is not effective in overall clinical improvement of COVID-19 patients. Other considerations are the time (when to start) and the duration of the intravenous treatment of the ascorbic acid therapy. Data on this particular information are lacking. The available trials have a few limitations. These are small sample sizes, the absence of data at the baseline, and the lack of continuous monitoring of serum ascorbic acid concentrations. Based on the current literature, there is a lack of understanding of whether the effect of intravenously administered ascorbic acid on lung function in COVID-19 patients is dependent on the patient’s initial ascorbic acid status upon admission (deficient versus sufficient). The lack of assessment of viral load in most studies made it difficult to understand the direct antiviral activity of ascorbic acid against SARS-CoV-2. Moreover, most of the studies did not measure the anti-oxidative biomarkers. Importantly, evidence from a small patient population may not have enough power to demonstrate the true effect of ascorbic acid on lung function-related outcomes. Furthermore, the variability of the dose of intravenous ascorbic acid made it difficult to compare the outcomes of various trials. More randomized, double-blind, clinical trials with greater patient populations are required to confirm the understanding of the effect of ascorbic acid on critically ill COVID-19 patients. In addition, studies are needed to determine the effective dose of intravenous ascorbic acid at which the lung function markers are improved without the deleterious effects in COVID-19 patients. In future studies, the vaccination status of the patients might be an important factor to consider.

## Figures and Tables

**Table 1:** Summary of human clinical trials investigating the effect of high-dose intravenous ascorbic acid on lung function markers of patients with severe COVID-19 infection ^1^.

Reference	Study Design and *n* (I/C)	Dose and Duration of Intravenous Ascorbic Acid	Drug Therapy	Outcome Measurements on Lung Function	Findings Related to Lung Function ^2^	Other Findings ^2^
High Dose Ascorbic Acid Studies						
[33]	Multicenter RCT *n* = 56 (27/29)	24 g/d for 7 d	Oseltamivir and Azithromycin. Heparin as needed	IMVFD-28 IMV days to day 28 HFNC days to day 28 NIV days to day 28 PiO_2_/FiO_2_ ratio Oxygen support	↑PaO_2_/FiO_2_(P/F) (*p* = 0.01)	Decreased IL-6 (*p* = 0.04) Total bilirubin (*p* = 0.03)
[78]	Retrospective cohort *n* = 76 (46/30)	12 g/d IV on day 1, then 6 g/d for 4 d	Standard therapy according to the Chinese National Health and Health Commission Office	Oxygen support status	↑Oxygen support status (≈64%) (improved lung function)	↑28-d mortality (*p* = 0.037)
Medium/Low Dose Ascorbic Acid Studies						
[79]	Prospective open-label RCT *n* = 60 (30/30)	2 g/d for 5 d	Lopinavir/Ritonavir and Hydroxychloroquine	RR SpO_2_	↑3rd day SpO_2_ (*p* = 0.014)	Longer hospitalization (*p* = 0.028) ↑3rd day temperature (*p* = 0.001) ↑Myalgia (*p* < 0.001) ↑Fever (*p* = 0.002)
[80]	Single-center, open-label, parallel-group RCT *n* = 20 (10/10)	Total dose: 8 g IV ascorbic acid for 10 d	24 mg oral melatonin and 200 mg oral zinc sulfate	PaO_2_/FiO_2_ ratio SaO_2_	No significant difference	No significant difference
[34]	Retrospective cohort *n* = 323 (153/170)	2 g/d IV started on day 3 following admission until discharge	Not reported	SpO_2_ at hospitalization	No significant change	↑Ferritin (*p* = 0.006) ↑CRP (*p* < 0.001)
[35]	Prospective, open-label RCT *n* = 150 (75/75)	50 mg/kg/d throughout hospitalization	Antipyretics, Dexamethasone, and prophylactic antibiotics	Need for ventilation Oxygen saturation Respiratory rate	No significant change	↑Clinical symptoms (*p* < 0.0001) ↑Hospital stay (*p* < 0.0001)

^1^ Abbreviations: CRP, C-reactive protein; HFNC, High flow nasal cannula; I/C, Intervention/Control (sample size); IL, Interleukin; IMV, Invasive mechanical ventilation; IMVFD-28, Invasive mechanical ventilation-free days in 28 days; IV, Intravenous; NIV, Non-invasive mechanical ventilation; PiO_2_/FiO_2_: Partial pressure of oxygen to the fraction inspired oxygen; RCT, Randomized controlled trial; RR, Respiration rate; SaO_2_, Blood oxygen saturation; SpO_2_: Peripheral capillary oxygen saturation. ^2^ ↑ Increased.
